# Psychometric Evaluation of the Serbian Adaptation of the Presentation of Online Self Scale (POSS) and Further Construct Validation

**DOI:** 10.5964/ejop.15483

**Published:** 2025-08-29

**Authors:** Bojana Bodroža, Chris Fullwood, Bojana M. Dinić

**Affiliations:** 1 Department of Psychology, Faculty of Philosophy, University of Novi Sad, Novi Sad, Serbia; 2 College of Psychology, Birmingham City University, Birmingham, United Kingdom; Stanford University, Stanford, CA, USA

**Keywords:** self-presentation, impression management, online presentation, validity, social media

## Abstract

The Presentation of Self Scale (POSS) was designed to measure four aspects of online self-presentation behaviour: Ideal self, Multiple selves, Consistent self, and Online presentation preference. Very few scales have been developed to measure online-self presentation attitudes and behaviour in Serbia. Thus, there is a need to validate a Serbian language version of the POSS to support further investigation of an increasingly ubiquitous aspect of the daily lives of Serbian people. This study aimed to examine psychometric properties of the POSS in the Serbian context i.e., its reliability, factor validity, and construct validity. The study was conducted on a sample of 360 adults. The four-factor model was confirmed, and it is invariant across genders. The Ideal self, Multiple selves, and Online presentation preference scales converge and show a similar pattern of relationships with validity variables, with Ideal self and Multiple selves showing high profile similarity. These three scales are associated with less sensitivity to the expressive behaviour of others, greater fear of negative evaluation, social media addiction, anxiety, lower self-esteem, and less loneliness. On the other hand, the Consistent self-scale is generally unrelated to the other POSS scales and correlates with better sensitivity to the expressive behaviours of others, less fear of negative evaluation, but greater loneliness. The POSS proved to be useful for examining self-presentation behaviours in the Serbian cultural context. The study revealed two main self-presentational patterns: one that is inauthentic and facilitated by the features of online communication and the other that is authentic and related to better social sensitivity.

Communicating online differs from face-to-face communication in a variety of meaningful ways. While not all online communication platforms should be considered equal, there are numerous features, or affordances, broadly associated with Internet-mediated forms of communication that have been shown to influence self-disclosure and self-presentation behaviour (Schlosser, 2020). In some instances, these affordances (e.g., perceived anonymity) may encourage deeper and broader self-disclosures (Clark-Gordon et al., 2019), while other features (e.g., magnified audience feedback) may lead to more idealised self-presentation (Manago et al., 2008). There has been considerable debate and academic interest in which, if any, of these features might facilitate inauthentic self-presentation, and the extent to which people present their ‘true’ selves while they are online (Fullwood, 2019; Schlosser, 2020). Such knowledge is not only of theoretical interest but may also be applied to understanding behaviour in various real-world contexts where it is important to gather an accurate impression of another person, for example during online dating or when using online platforms as screening and selection tools during job recruitment. Roulin and Levashina (2019) found that LinkedIn was an effective tool for accurately assessing an applicant’s skills, personality and cognitive ability. The professional nature of the site, as well as the potential for one’s networks to be tied to existing offline contacts, might encourage users to present themselves authentically (Haimson et al., 2021). Online dating, on the other hand, may be associated with an increased potential for inauthentic self-presentation, and this may in part be explained by increased competition and motivation to attract other daters (Peng, 2020). Thus, there is a complex nexus between the affordances the medium offers to users and the individual’s motivations for using the medium in determining self-presentation behaviour.

Self-presentation may be seen as “the goal-directed activity of controlling information to influence the impressions formed by an audience about the self” (Schlenker & Wowra, 2003, p. 871). Because there are numerous personal, material, occupational and social rewards associated with leaving others with desired impressions, self-presentation is usually a daily concern for most people (Leary & Allen, 2011). With the proliferation of information and communication technologies over the past few decades, as well as an increase in the amount of time people are spending online, self-presentation concerns have arguably become more pervasive, with the potential to reach and influence a much wider audience than previously possible (Fullwood, 2019). Understanding how different Internet-mediated communication tools affect how we relate to and present ourselves to others is thus a topic of significant psychological interest. While observing people’s online conduct may provide a window into their self-presentation behaviour, many individuals prefer to remain anonymous online (Kang et al., 2013), and there are significant ethical and methodological challenges for researchers associated with linking online behaviour to psychological constructs of interest (Kraut et al., 2004). Thus, there is a need to establish reliable and valid self-report measures which tap into online self-presentation behaviours and attitudes.

One such measure is the Presentation of Online Self Scale (POSS) (Fullwood et al., 2016). The development of the POSS was underpinned by Walther’s (1996) and Walther and Parks (2002) hyperpersonal theory of computer-mediated communication and Goffman’s (1959) notion of the performed self. The basic premise of Goffman’s self-presentation theory is that people are highly motivated to control the impressions others form of them and project desirable self-images. Using a dramaturgical analogy, Goffman proposed that, as social actors, we are attuned to our audiences and adjust our performance accordingly, and this often involves accentuating positive aspects of the self while suppressing more negative aspects. Hyperpersonal theory (Walther, 1996; Walther & Parks, 2002) argues that technology-mediated forms of communication provide individuals with numerous unique affordances, which permit greater control over the information they present to others, potentially resulting in more optimal forms of self-presentation. These affordances, though not necessarily associated with all Internet-mediated communication platforms, include anonymity (others do not know who I am, so I can be who I want to be), invisibility (others cannot see me, so I do not have to worry about how I come across), asynchronicity (as some interactions are not in real-time, I have more time to think about what I am saying), and the reallocation of cognitive resources (with fewer communication cues to attend to, I can put all my efforts into presenting myself more optimally). As an example, when individuals are anonymous, they may feel less inhibited about disclosing certain types of information (e.g., private or personal) that they might find more difficult revealing face-to-face. Indeed, Lapidot-Lefler and Barak (2015) found that participants disclosed more emotion during dyadic interactions when they were anonymous (i.e., using random aliases over real names) and invisible to their chat partner. Conversely, there is also evidence that social media users are more likely to communicate in a manner that reflects their actual rather than idealised personality traits when they are identifiable (Back et al., 2010).

The POSS measures the degree to which individuals perceive their online self-presentation to be idealised (Ideal self); the extent to which we show different sides of ourselves across multiple online platforms (Multiple selves); the degree to which online and offline self-presentations are similar (Consistent self); and an individual’s preference for presenting the self online over face-to-face (Online presentation preference). To date, the POSS has been mostly tested in the UK (Fullwood et al., 2016; Fullwood et al., 2020; Wilson et al., 2024), the USA (Fullwood et al., 2020; Mann & Blumberg, 2022), and Australia (Fullwood et al., 2020). However, when applied to adults in Ireland, results showed that one item (15) should be excluded due to low factor loading and that the optimal is the 3-factor solution with factors adaptable self, authentic self, and freedom of self online (Strimbu et al., 2021). Furthermore, its use in non-English speaking countries has revealed some issues with factor structure. For example, in a Turkish sample in which the Multiple Selves Scale was not used, model fit testing suggested the exclusion of three items in order to achieve a good model fit (Güler et al., 2022). When used on an Indonesian sample, three items were deleted due to low item-total correlation (Sari & Sa’id, 2023), but no test of factor analysis was provided. Factor structure was not tested in other studies using Indonesian samples (Candra et al., 2023; Sa’diyah & Fauziyah, 2021). In addition, no test of factor structure was provided in a Russian sample (Rubtsova et al., 2021).

In this study, we explore the construct validity of the POSS in a sample of Serbian social networks users. According to Datareportal’s ‘Digital in Serbia’ 2024 report, approximately 90% of the Serbian population were online at the start of 2024, with around 5 million, or 70.3%, of the total population using social media (Kemp, 2024). The current Internet penetration rate in Serbia represents a 20% increase from January 2020 (Kemp, 2024). In comparison, in the UK around 98% of the population were using the Internet in 2024, but this was only a 3% increase from 2020 (Statista, 2024). Alongside the recent rapid growth in Internet use in Serbia, there has been increasing academic interest in how being online affects the self-presentation behaviour of Serbian people. Goljović (2024) for example investigated the link between smartphone addiction and perfectionist self-presentation behaviour. Yet while numerous measures of general self-presentation behaviour have been adapted for the Serbian context (e.g., Goljović, 2024; Simunović & Žeželj, 2023), there exist few measures in the Serbian language which focus on online-specific behaviour. Notable exceptions include Majstorović et al.’s (2024) adaptation of the Self-Presentation on Facebook Questionnaire (Michikyan et al., 2015) and Jovanović et al.’s (2023) Psycho-Social Aspects of Facebook Use (PSAFU) scale, which includes a self-presentation subscale. The use and application of these scales, however, are more limited in the online context as they focus exclusively on Facebook and are thus unsuitable for investigating broader self-presentation attitudes and behaviour across varied technology-mediated communication platforms.

Previous research has uncovered several associations between the four subscales of the POSS and various theoretically related psychological constructs and individual difference factors, supporting the measure’s construct validity. Ideal self has been found to be more pronounced among female rather than male adolescents (Candra et al., 2023), which may be a consequence of greater pressure for female sexual objectification and heightened social comparison with other females (Manago et al., 2008). Ideal self is also associated with lower self-concept clarity (Fullwood et al., 2016; Fullwood et al., 2020), being younger, and more socially anxious (Fullwood et al., 2020). These traits are often linked to lower levels of self-esteem (Campbell, 1990; Fatima et al., 2017; Meier et al., 2011) and thus idealised online self-presentation may be a compensatory behaviour to improve self-worth (Fullwood et al., 2016). Collectively, these findings support the poor-gets-richer hypothesis (or social compensation model), which argues that individuals may make use of the affordances of online communication to offset any social deficits, for example poorer social networks, experienced in their offline lives (Kraut et al., 2002; McKenna & Bargh, 1998). Indeed, Mehdizadeh (2010) found that people with lower self-esteem produced more self-promotional content on their social media profiles.

Multiple selves is associated with lower levels of self-concept clarity (Fullwood et al., 2016; Fullwood et al., 2020). Low self-concept clarity is associated with identity uncertainty, and thus more varied self-presentation behaviours may reflect a level of doubt about how to present the self to others. Alternatively, this behaviour may represent a form of self-exploration to help resolve an identity crisis (Fullwood et al., 2016). Using an adapted version of the POSS (POSSA, Strimbu et al., 2021), Nitschinsk et al. (2022) found that adaptable self-presentation, which is conceptually similar to Multiple selves, was associated with Machiavellianism and psychopathy, suggesting that it might also be understood as a manipulative strategy. Multiple selves is also associated with engaging in less frequent self-monitoring and having lower self-esteem (Fullwood et al., 2020). Low self-monitors manifest more authentic self-presentation and are less likely to adjust their self-presentation based on audience reactions (Snyder, 1987). It may be that the affordances of online communication channels permit low self-monitors to behave more spontaneously and with less inhibition. Those with lower self-esteem, however, may cater their self-presentation styles to different audiences in a bid for approval (Fullwood et al., 2020). Wilson et al. (2024) also found a link between Multiple selves and self-oriented perfectionism. As with the ideal self, perfectionists may value the online world for providing them the opportunity to present a more perfect version of the self to others, while simultaneously masking their imperfections (Wilson et al., 2024).

Consistent self has been associated with having higher self-esteem, engaging with social media less often (Mann & Blumberg, 2022), better wellbeing (Güler et al., 2022) and higher self-concept clarity (Fullwood et al., 2016; Fullwood et al., 2020). Overall, these findings suggest that those with more positive self-regard are more likely to present a version of the self online which is analogous with their offline self-presentation. Conversely, Online presentation preference has been associated with having poorer wellbeing (Güler et al., 2022), lower self-concept clarity (Fullwood et al., 2016), lower self-esteem and higher social anxiety (Fullwood et al., 2020), once more supporting the social compensation model (Kraut et al., 2002; McKenna & Bargh, 1998). Additionally, high self-monitors display a preference for communicating offline, whereas low self-monitors prefer online interaction (Fullwood et al., 2020). It is not surprising that high self-monitors prefer to communicate with others offline, as they may feel that this allows them to more easily read the reactions of their audience, for example because they have access to a greater variety of communication cues (e.g., non-verbal communication), which may be missing or attenuated in many online platforms.

So far, self-presentation, as measured by POSS, has not been studied in relation to social anxiety, social media addiction or loneliness — widely-researched negative correlates of social media use in general. However, a number of studies have investigated these relationships using other measures of self-presentation. Bodroža and Jovanović (2016) showed that, across a wide range of social media behaviours, social anxiety was most strongly related to self-presentational behavioural patterns, specifically efforts to present oneself in a positive or idealized fashion. Additionally, the more individuals self-presented on social media, the more they exhibited problematic or addictive patterns of social media use. Zahra and Muhammad (2024) confirmed that inauthentic self-presentation is indeed related to social anxiety, while Monacis et al. (2021) provided evidence that presenting a more idealised version of the self on social media fuels addiction, perhaps because it makes people feel good about themselves. Based on these findings, it seems that inauthentic (whether idealized or multiple) self-presentation could be understood as a defensive strategy to protect one’s sense of self-worth, whereby presenting a polished version of the self, individuals are shielding themselves against criticism. However, such a defence may come at the price of social media addiction.

Regarding self-presentation in relation to social functioning and adaptation, studies have shown that authentic and honest self-presentation could lead to better perceived social support, which could further benefit an individual’s well-being (Gao et al., 2023; Kim & Lee, 2011; Wang et al., 2019). Studies have also shown that people who score higher on loneliness use social media to compensate for what they lack from offline relationships (see O’Day & Heimberg, 2021). Based on all these findings, we could indirectly infer that authentic and consistent online-offline self-presentation would be related to less loneliness, but also that lonely individuals would prefer online presentation. However, the direct relationship between the different forms of self-presentation and loneliness remains unaddressed and is yet to be studied. The aim of this study was to explore psychometric characteristics of the Serbian adaptation of the Presentation of Online Self Scale (POSS). More precisely, the originally proposed four-factor model was tested via confirmatory factor analysis, as well as gender invariance in order to ensure that the items have the same meaning across genders. Based on previous research findings, we make a number of predictions for potential associations between the four POSS subscales and numerous theoretically related variables:

**H1**: Ideal self: We expect *Ideal self* to be associated with, a) being female (Candra et al., 2023), b) younger (Fullwood et al., 2020), c) greater social anxiety (Zahra & Muhammad, 2024), and d) social media addiction (Monacis et al., 2021).
**H2:** Multiple selves: We expect *Multiple selves* to be associated with, a) lower self-monitoring (Fullwood et al., 2020), b) lower self-esteem (Fullwood et al., 2020), and c) social media addiction (Monacis et al., 2021).
**H3**: Consistent self: We expect *Consistent self* to be associated with, a) higher self-esteem (Güler et al., 2022; Mann & Blumberg, 2022), and b) higher self-monitoring (Fullwood et al., 2020).
**H4**: Online presentation preference: We expect *Online presentation preference* to be associated with, a) lower self-monitoring (Fullwood et al., 2020), b) lower self-esteem (Fullwood et al., 2020), and c) social media addiction (Monacis et al., 2021).

While no previous literature has tested relationships between POSS subscales and loneliness, it was shown that authentic and consistent online-offline self-presentation could benefit social functioning (Gao et al., 2023; Kim & Lee, 2011; Wang et al., 2019). Additionally, people who score higher on loneliness have been consistently shown to use social media to address unfulfilled needs from their face-to-face relationships (see O’Day & Heimberg, 2021), which might suggest that communication in an online setting could be especially attractive for them. Based on this, we would expect loneliness to positively correlate with *Online presentation preference* (H4d) and negatively to *Consistent self* (H3c). Furthermore, while no previous literature is available to guide specific predictions relating to the POSS subscales and generalised anxiety, Muzaffar et al. (2018) found an association with increased and repetitive Facebook behaviour, thus we deemed its inclusion warranted.

## Method

### Sample

The sample comprised 360 adults (61.2% identified as women) from the Serbian general population active online, aged 18 – 56 (*M* = 22.60, *SD* = 6.01). The majority of participants were students (70.4%), with 18% having finished elementary or high school, 1.9% finishing college, and 9.1% having a university bachelor’s or master’s degree. Data were collected online as a part of students’ pre-exam activities. Each student collected data (i.e., sent a link to the instruments) from 5 participants, ensuring sex and age balance. Informed consent was obtained from all individual participants included in the study. The study was approved by the Ethics Committee for Psychological Research, Faculty of Philosophy in Novi Sad.

### Instruments

The Presentation of Online Self Scale (POSS; Fullwood et al., 2016) comprises 21 items with a 5-point response scale (from 1 = Strongly disagree to 5 = Strongly agree), measuring 4 aspects of online self-presentation behaviour: Ideal self (9 items), Multiple selves (5 items), Consistent self (4 items), and Online presentation preference (3 items).

The back-translation method was used to ensure the correct meaning of the scale (Hedrih, 2020). In the first instance, after the preparation and contacting the author of the scale, BD translated the POSS from English to Serbian. This was followed by a back-translation of the scale to English by BB, and a comparison between the original and back-translated scale by the author of the scale, CF. Then this version was evaluated by 50 psychology students to check understandability, interpretation, and cultural relevance of the translation. In line with this cognitive debriefing, some terms were rephrased and updated to be more appropriate to Serbian language (e.g., instead of “real life”, we used “who I really am”, instead of “offline”, we used “in person”). The proposed changes were again reviewed by CF and proofread. The final version of the Serbian adaptation can be seen in the Appendix.

Revised Self-monitoring Scale (Lennox & Wolfe, 1984). This scale consists of thirteen 6-point scale items (from ‘0 = Certainly, always false’ to ‘5 = Certainly, always true’) measuring Ability to modify self-presentation (7 items) or Sensitivity to expressive behaviour of others (6 items). As Croatian and Serbian languages are considered different national variants and official registers of the pluricentric Serbo-Croatian language (Šipka, 2019), we used the Croatian translation of the scale (Burušić, 2003) with slight wording modifications. The calculated model fit for proposed 2-factor structure and fit indices were good (DWLSχ^2^(64) = 101.10, CFI = .98, TLI = .97, RMSEA = .04, 90% CI [.02, .06], SRMR = .06) and significantly better than one-factor solution (Δχ^2^(1) = 110.11, *p* < .001). Correlation between factors was .58.

Brief Fear of Negative Evaluation Scale (Brief-FNE: Leary, 1983, for Serbian adaptation see Ranđelović & Želeskov Đorić, 2017). This scale consists of twelve 5-point scale items (from ‘1 = Not at all characteristic of me’ to ‘5 = Extremely characteristic of me’) measuring social anxiety.

Bergen Social Media Addiction Scale (BSMAS; Andreassen et al., 2012, for Serbian adaptation see Dinić et al., 2021). BSMAS consists of six 5-point scale items (from ‘1 = Very rarely’ to ‘5 = Very often’), each referring to criteria for social media addiction.

Single-item self-esteem scale (Robins et al., 2001; for Serbian adaptation see Dinić & Branković, 2022). It consists of one item (“I have high self-esteem”) on a 7-point response scale (from ‘1 = Not very true of me’ to ‘7 = Very true of me’).

Short De Jong Gierveld Loneliness Scale (Short DJGLS; De Jong Gierveld & Van Tilburg, 2006, for Serbian adaptation see Jovanović & Žuljević, 2013). This scale comprises six binary items (Yes/No) referring to emotional or social loneliness.

Generalized Anxiety Disorder-7 (GAD-7; Spitzer et al., 2006, for Serbian adaptation see Rokvić, 2019). GAD-7 consists of seven 4-point scale items (from ‘0 = Not at all’ to ‘3 = Nearly every day’) measuring symptoms of generalised anxiety disorders (GAD) over the past two weeks.

Descriptives and reliabilities for all used instruments are presented in Table 1.

**Table 1:** Construct Validity Correlations of the Serbian Adaptation of the Presentation in Online Self Scale (POSS)

Scale/Variable	Ideal self	Multiple selves	Consistent self	Online presentation preference	*M* (*SD*)	α
Ideal self	1				1.93 (0.72)	.77
Multiple selves	.56** (0.14)	1			1.36 (0.56)	.74
Consistent self	-.03 (0.62)	-.14** (0.30)	1		3.45 (0.10)	.65
Online presentation preference	.50** (0.25)	.31** (0.25)	.003 (0.78)	1	1.82 (0.73)	.44
Ability to modify self-presentation	.09	.04	.03	-.11*	2.79 (0.91)	.75
Sensitivity to expressive behaviour of others	-.03	-.10*	.25**	-.17**	3.54 (0.89)	.79
Fear of negative evaluation	.25**	.10*	-.13*	.25**	2.95 (0.86)	.89
Social media addiction	.37**	.19**	-.03	.22**	2.51 (0.93)	.79
Self-esteem	-.21**	-.06	.04	-.29**	5.27 (1.56)	-
Loneliness	-.17**	-.06	.11*	-.19**	2.30 (0.49)	.69
General anxiety disorder	.11*	.08	-.07	.16**	1.31 (0.84)	.91
Age	-.12*	-.05	-.04	.05	22.60 (6.01)	-

*Note*. Profile (dis)similarity *D* was in parentheses.

*p < .05. **p < .01.

### Data Analysis

The factor models were tested via confirmatory factor analysis. Due to the violation of multivariate normality, a diagonally weighted least squares (DWLS) estimator was used. Model fit was evaluated via values ≥ .90 for comparative fit index (CFI) and Tucker-Lewis Index (TLI), as well as ≤ .08 for Root Mean Square Error of Approximation (RMSEA) and Standardized Root Mean Square Residual (SRMR), which refer to acceptable model fit (Hu & Bentler, 1999). For measurement invariance across genders, configural (equal factor structure), metric (equal loadings), and scalar invariance (equal thresholds) were tested. A difference in CFI (ΔCFI) ≤ .01 and a difference in RMSEA (ΔRMSEA) ≤ -.015 were considered indicative of nonsignificant differences between the models (Cheung & Rensvold, 2002). All models were tested in JASP 0.19.0 (JASP Team, 2024). Gender differences were tested via *t*-tests and Cohen’s *d* was provided as an effect size measure. Construct validity correlations were tested via Pearson correlation coefficients. Profile (dis)similarity between the POSS scales, based on correlations with validity variables, was calculated using Cronbach and Gleser’s (1953) *D* statistic. Lower *D* values indicate smaller distances and higher similarity. *D* could be interpreted in terms of Cohen’s *d* as an effect size measure (small 0.2, medium 0.5, and large 0.8). The data is available at Bodroža et al. (2025). The data for this study are part of a larger project and all data from the project are available at Bodroža et al. (2025). The study was not preregistered.

## Results

### Confirmatory Factor Analysis

**Figure 1:**
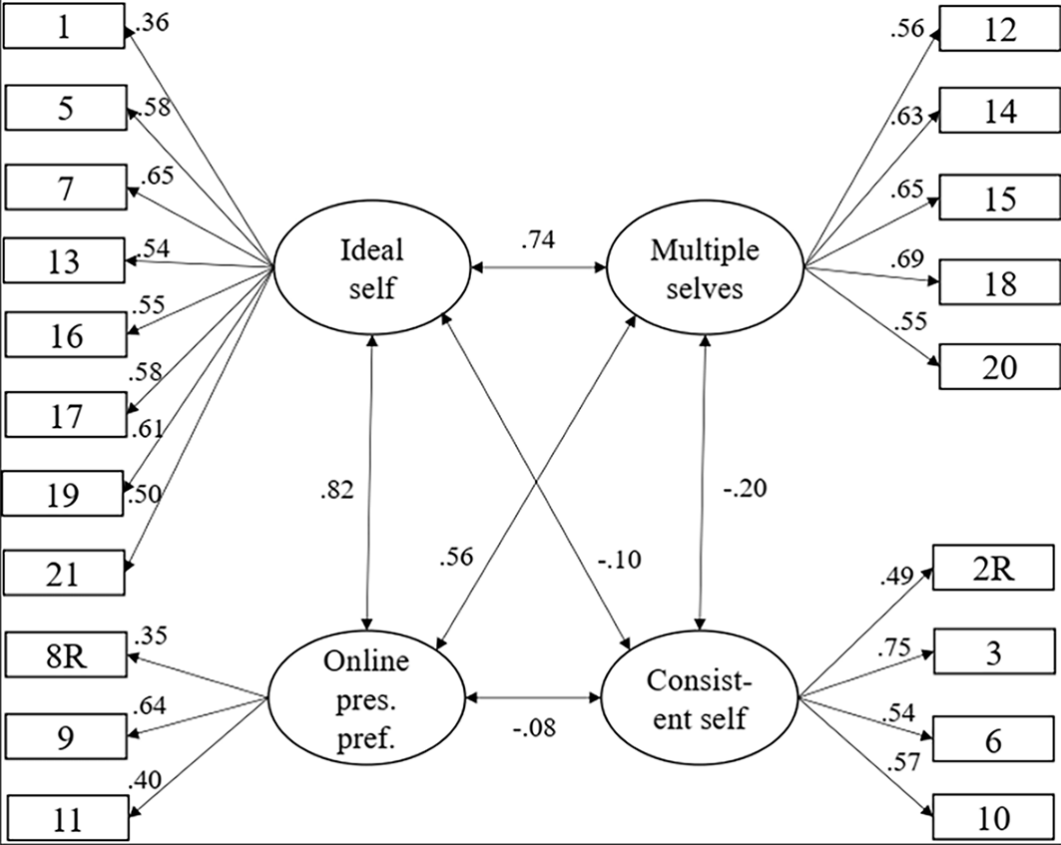
Model Parameters for the 4-Factor Model of the Serbian Adaptation of the Presentation in Online Self Scale (POSS) *Note*. Items were recoded before analysis.

The originally proposed 4-factor model had marginal model fit (DWLSχ^2^(183) = 513.50, *p* < .001, CFI = .88, TLI = .86, RMSEA = .07, 90% CI [.06, .08], SRMR = .09). Based on modification indices, only when residual correlations between Items 4 and 3, and 4 and 2 were included, model fit was satisfactory (DWLSχ^2^(181) = 419.28, *p* < .001, CFI = .91, TLI = .90, RMSEA = .06, 90% CI [.05, .07], SRMR = .08). However, since these items are from different scales and considering that item 4 emerged in both combinations, we deleted Item 4 from the final solution. The final solution had good fit indices (DWLSχ^2^(164) = 312.51, *p* < .001, CFI = .94, TLI = .93, RMSEA = .05, 90% CI [.04, .06], SRMR = .07). Factor loadings ranged from .35 to .75 and correlations between factors from -.20 to .82 (Figure 1). Omegas were .74 (Ideal self), .73 (Multiple selves), .67 (Consistent self), and .41 (Online presentation preference).

Considering relatively high correlations between 3 factors (Ideal self, Multiple selves, and Online presentation preference), we tested a hierarchical model with 4 first-order factors and one second-order factor consisting of the three first-order factors. This model had comparable CFI (ΔCFI = -.004) and RMSEA (ΔRMSEA = .002) fit indices to the 4-factor model (DWLSχ^2^(166) = 325.06, *p* < .001, CFI = .93, TLI = .92, RMSEA = .05, 90% CI [.04, -.06], SRMR = .07), although Δχ^2^(2) = 12.55 was significant at *p* < .002, but nonsignificant at *p* < .001 level. Loadings on the first-order factors ranged from .35 to .74, while loadings on the second-order factor were high, .98, .76, and .80, respectively. Correlation between the second-order factor and Consistent self was -.15.

We also tested the 2-factor model with items from the scales Ideal self, Multiple selves, and Online presentation preference merged into one factor and Consistent self scale representing a separate factor and this solution showed worse fit indices compared to the hierarchical model, with ΔCFI = -.011, ΔRMSEA = .003, and Δχ^2^(3) = 28.8, *p* < .001 (DWLSχ^2^(169) = 353.86, *p* < .001, CFI = .92, TLI = .91, RMSEA = .06, 90% CI [.05, .06], SRMR = .08). Considering equally well fit indices of the 4-factor model and the hierarchical model, we decided to further examine the 4-factor model, as it allows us to compare our results with the results of previous studies. Additionally, we tested a reduced, 3-factor model proposed by Strimbu et al. (2021) but the model did not converge.

### Measurement Invariance Across Genders

Results of measurement invariance testing showed that there is scalar invariance between genders, considering that ΔCFI and ΔRMSEA did not reach the thresholds for significantly worse model fit in scalar compared to the rest of the invariance levels (Table 2). Therefore, gender comparisons are justified.

**Table 2:** Measurement Invariance of the Serbian Adaptation of the Presentation in Online Self Scale (POSS) Model Across Genders

Level	DWLSχ^2^(*df*)	CFI	TLI	RMSEA 90% CI	SRMR	ΔCFI	ΔRMSEA
Configural	433.63(328)	.95	.95	.04 [.03, .05]	.09	–	–
Metric	476.74(344)	.94	.94	.05[.04, .06]	.09	.01	.003
Scalar	492.92(360)	.94	.94	.05[.04, .06]	.09	.00	.001

*Note.* All χ^2^s were significant at *p* < .001.

Considering that scalar invariance had been achieved, gender differences were tested. The only significant and small differences were found in Multiple selves, with men scoring higher than women (Table 3).

**Table 3:** Gender Differences in the Serbian Adaptation of the Presentation in Online Self Scale (POSS) Scales

	Men (*n* = 139)		Women (*n* = 221)		Gender comparisons	
POSS scales	*M*	*SD*	*M*	*SD*	*t*(358)	*d*
Ideal self	1.98	0.68	1.89	0.73	1.12	0.12
Multiple selves	1.44	0.59	1.30	0.52	2.37*	0.26
Consistent self	3.44	1.01	3.46	0.92	-0.18	0.02
Online presentation preference	1.87	0.77	1.78	0.70	1.09	0.12

*p < .05

### Construct Validity Correlations and Alpha Reliabilities

Considering the number of items per scale, alpha reliabilities for the POSS scales were acceptable, except for Online presentation preference (Table 1). The POSS scales Ideal self, Multiple selves, and Online presentation preference are positively related, whereas Consistent self is only weakly negatively related to Multiple selves (Table 1). Regarding the Self-monitoring domains, the Ability to modify self-presentation formed a negative relation with Online presentation preference (supporting H4a), while Sensitivity to expressive behaviour of others was related positively to the Consistent self (supporting H3b) and negatively to Multiple selves (supporting H2a) and Online presentation preference (supporting H4a). Fear of negative evaluation of others (social anxiety) correlated positively to Ideal self (supporting H1c), Multiple selves (no prediction made), and Online presentation preference (no prediction made), and negatively to Consistent self (no prediction made). Social media addiction also correlated positively to Ideal self (supporting H1d), Multiple selves (supporting H2c), and Online presentation preference (supporting H4c). Lower Self-esteem was related to Ideal self (no prediction made) and Online presentation preference (supporting H4b). Loneliness was related to Ideal self (no prediction made) and Online presentation preference (supporting H4d), while hypothesized negative correlation with consistent self (H3c) was not observed. Generalised anxiety disorder was positively related to Ideal self (no prediction made) and Online presentation preference (no prediction made), while Consistent self had a positive correlation with Loneliness (no prediction made). Finally, younger individuals showed a preference for presenting the Ideal self online (supporting H1b).

Profile (dis)similarity showed that differences in validity correlations (all presented in Table 1, except age) were negligible between Ideal self and Multiple selves, small between Online presentation preference on the one side and Ideal self and Multiple selves on the other, as well as between Multiple selves and Consistent self, while they were medium for comparison between Consistent self on the one side and Ideal self and Online presentation preference on the other side. Thus, Ideal self and Multiple selves showed high profile similarity^1^1Considering the results of profile similarity, we tested a hierarchical model with 4 first-order factors and a second-order factor consisting of the Ideal self and Multiple selves. The model fit was: DWLSχ^2^(165) = 324.31, *p* < .001, CFI = .93, TLI = .92, RMSEA = .05, 90% CI [.04, .06], SRMR = .07, with ΔCFI = -.004 and ΔRMSEA = .002 that showed no difference in comparison to 4-factor model (but Δχ^2^(1) = 11.8 was significant at *p* < .001). However, the correlation between second-order factor and Online presentation preference was high, .81, pointing, again, that second-order factor with Online presentation preference included would be more appropriate. In addition, 3-factor model with merged items from Ideal self and Multiple selves showed worse mode fit: DWLSχ^2^(167) = 348.53, *p* < .001, CFI = .92, TLI = .91, RMSEA = .06, 90% CI [.05, .06], SRMR = .08., while there are small to medium differences between the rest of the scales considering tested validity correlations.

## Discussion

The results of this study confirm the factor validity of the Serbian adaptation of the Presentation of Online Self Scale, after slightly adjusting item formulations and removing one item. The mean factor loading was .56, which is comparable to original research (.65 in Fullwood et al., 2016). Although in the original research the lowest loading was .43, in this Study 2 items had loading below .40 (1 and 8), but still significant and above .33, which is the recommended cut-off (Tabachnick & Fidell, 2019). Considering the number of items, it could be concluded that the scales showed adequate alpha and omega reliability, with alphas in line with previous research (Fullwood et al., 2016, 2020). However, the alpha and omega of the scale Online presentation preference is somewhat below acceptable level. This issue could partly be attributed to a low number of items (only 3). Additional analysis suggested that items in this scale are somewhat difficult and had low variability, meaning that there is a stronger preference for online compared to offline communication in the sample. Future studies should address this issue either by adding more items or by rephrasing the existing ones.

Intercorrelations of the factors suggest that three factors — Ideal self, Multiple selves, and Online presentation preference converge, whereas the Consistent self is relatively independent. The alternative model with a second-order factor consisting of these three factors showed comparable model fit. However, the profile similarity index showed that only Ideal self and Multiple selves showed high similarity, while Online presentation preference showed a somewhat distant profile of validity correlations. We should also note that model with a second-order factor consisting of Ideal self and Multiple selves showed a very high correlation between this second-order factor and Online presentation preference. Therefore, although correlations between latent factors can be considered as high, correlations based on average scores, which are usually used, showed the distinction between Online presentation preference and the other two factors. Analysis of the Ideal Self scale items reveals a strong emphasis on the possibility of being different, with only one item explicitly referencing an ideal version of the self. This suggests that the scale's core content may primarily revolve around divergence from the real self, potentially reflecting idealised self-presentation. Consequently, the observed high convergence with the Multiple Selves scale is unsurprising. Presenting multiple identities online can be a means of exploring diverse self-presentational possibilities, including idealised ones. However, this finding necessitates a more nuanced conceptual distinction within the Ideal Self scale.

As previously noted, high to moderate correlations emerged between the Ideal Self, Multiple Selves, and Online Presentation Preference scales, contrasting with the largely independent Consistent Self scale. This suggests the existence of two distinct patterns of online self-presentation. The first pattern, characterized by inauthenticity, involves variations in idealised or multiple identities that diverge significantly from the perceived real self. This pattern is often associated with a preference for online interactions. The second pattern is characterized by a more realistic online identity, converging with an individual's self-perception and offline presentation. However, the low negative correlation between these two broad patterns (or zero to low correlations between the three scales and Consistent Self) indicates that they are not mutually exclusive. Most individuals likely engage in both types of online self-presentation, contingent upon the specific context and their goals. Results showed that the proposed four-factor solution is invariant across genders, justifying further gender comparisons. Inconsistently with H1a and previous research (Candra et al., 2023), women did not self-present in a more idealistic manner compared to men. Contrary to expectations, men preferred multiple self-presentations over different platforms more than women. It may be that, for the Serbian men in this sample, presenting multiple selves is a useful strategy when flirting or seeking intimate partners online, and indeed these are behaviours more often practised by men (e.g., Rice et al., 2015). This is consistent with the conclusion that multiple self could serve as a manipulative strategy, as it is closely related to socially aversive or dark traits (Nitschinsk et al., 2022), which are generally higher in men (e.g., Dinić et al., 2018). As this finding on gender differences in multiple self has not been found in previous research, this is however a tentative explanation, and we would recommend further investigation to shed light on this relationship. In line with previous findings (Fullwood et al., 2020) and H1b, idealistic self-presentation was also characteristic of the younger population. As suggested by Fullwood et al. (2020), it is likely that younger people may have a greater need to be liked by others.

In addition, correlations with construct validity variables were meaningful and largely consistent with theoretical expectations and our hypotheses. Individuals with an online presentation preference and those who engage in multiple online self-presentations tend to exhibit lower sensitivity to others’ expressive behaviours, therefore supporting H4a and H2a, however only online preference was also associated with an ability to modify self-presentation. Consistent self on the other hand was positively associated with sensitivity to the expressive behaviour of others, supporting H3b. Individuals who present idealised selves were more likely to fear negative evaluations by others, supporting H1c. Moreover, individuals who prefer online, idealised and multiple self-presentation demonstrate a more addictive pattern of social media use, supporting H1d, H2c, and H4c. Finally, those with a preference for presenting the self while online tend to have lower self-esteem, supporting H4b. This pattern of online self-presentational behaviour supports the Poor-Gets-Richer hypothesis or compensation theory (Kraut et al., 2002; Valkenburg et al., 2005) which states that poorly adapted individuals will be more attracted to online communication, as they feel protected by anonymity and possibility of careful crafting of the messages. These findings are also largely in line with the results of previous studies (e.g., Fullwood et al., 2020).

It should be noted, however, that some of our hypotheses were not supported, while we obtained some of the non-hypothesised relations. For example, we did not obtain a negative relation of Multiple selves with self-esteem (H2b), but we obtained negative correlations between idealised self-presentation and online presentation preference with self-esteem and loneliness, which was not hypothesised. These results indicate that low self-esteem individuals tend to present themselves in a slightly idealised manner, and perhaps prefer communicating online because of this, however they do not necessarily feel confident in presenting multiple identities. We also found that those who are more fearful of negative evaluations from others, prefer to communicate online and are more likely to present idealised and multiple self-presentations. This suggests that being online can shield people from negative evaluations, perhaps because one can hide one’s identity more easily or because there is greater control over which information one chooses to share, which aligns with the core tenets of hyperpersonal theory (Walther, 1996; Walther & Parks, 2002). It also appears that self-presentational strategies aimed at impressing others can be effective and beneficial, as individuals who employ them may experience reduced feelings of loneliness. This finding is comparable to the results of Błachnio et al. (2016) who found that the use of self-promotional self-presentation is negatively related to loneliness. Given the limited prior research on the relationship between loneliness and self-presentational tactics — especially those measured by the POSS, further investigation into these relationships is warranted.

On the other hand, Consistent self, i.e., realistic and congruent online/offline self-presentation is related to better social sensitivity, i.e., sensitivity to the expressive behaviours of others and less fear of others’ negative evaluation, but also to higher loneliness. Therefore, individuals who are not overly sensitive to negative evaluations but are sensitive to others’ communication style, tend to communicate honestly with others in the online environment, which corresponds with previous findings (Fullwood et al., 2020; Mann & Blumberg, 2022). However, it seems that expressing your true self online could bring unexpected perils, namely it could be linked to feelings of loneliness. This finding is opposite to the Hypothesis H3c and largely inconsistent to previous studies (Gao et al., 2023; Kim & Lee, 2011; Wang et al., 2019). Tentatively, this might suggest that people who express a consistent self are less prone to compromising and might expect that others take them as they are. As this is the only study so far to reveal such a relationship, replication of this finding is necessary.

The findings of this study are limited in several ways. First, our sample was 70% students, limiting our conclusions primarily to emerging educated adults. As the Online presentation preference scale had low alpha and omega, results regarding this aspect of self-presentation should be interpreted with caution. Future studies should widen the content and improve the reliability of this scale by adding new items. To date, no research has examined POSS longitudinally, leaving the temporal stability of online self-presentational behaviours uncertain. Furthermore, an individual's online self-presentation may vary across different platforms. Therefore, future research could investigate differences in POSS scores at a single platform over time, as well as across various online platforms, where we might anticipate diverse affordances and communication norms.

We argue that this work offers valuable support to Serbian scientists and practitioners by providing a psychometrically validated instrument in their own language to assess diverse forms of online self-presentation behaviours and attitudes. As social interaction increasingly shifts to digital platforms, the Serbian version of the POSS enables more nuanced investigation of a central and pervasive aspect of modern life. We anticipate that this tool will facilitate deeper understanding of the psychological implications of digital connectivity, including how online communication preferences may relate to experiences of loneliness, self-esteem, and broader social well-being. To conclude, the results of this study indicated that the POSS measure, after certain improvements, is an adequate and useful instrument for measuring the self-presentational behaviours of adult social media users. The self-presentational behaviours measured by the POSS could be brought down to two general patterns: one that is inauthentic and facilitated by the features of online communication, which is characteristic of less adapted individuals, and the other that is authentic and related to better social sensitivity.

## Supplementary Materials

**Table:** 

Type of supplementary materials	Availability/Access
Data	
a. Self-presentation - dataOSF.	Bodroža et al. (2025)
b. Excel codebook with data analysis coding terms.	Bodroža et al. (2025)
Material	
Output POSS.	Bodroža et al. (2025)

## Data Availability

The data that support the findings of this study are openly available on the Open Science Framework at Bodroža et al. (2025).

## Appendix

### The Serbian Adaptation of the Presentation of Online Self Scale (POSS)

Na sledeće stavke odgovorite tako što ćete označiti odgovor koji najbolje opisuje kako se osećate u onlajn okruženju.

1 – uopšte se ne slažem
2 – ne slažem se
3 – podjednako se i ne slažem i slažem
4 – slažem se
5 – potpuno se slažem
  1. Onlajn okruženje mi omogućava da se izrazim svoja razmišljanja i osećanja.
  2. Onlajn ne mogu da budem to što zaista jesam.
  3. Onlajn sam uvek onaj pravi ja (ona prava ja).
  4. Način na koji sebe predstavljam onlajn značajno se razlikuje od toga kakav sam zaista.
  5. Onlajn komunikacija mi omogućava da kažem stvari koje ne mogu uživo.
  6. Imam osećaj da je moja onlajn ličnost - pravo ja.
  7. Volim da budem onlajn jer mi to dozvoljava da budem drugačija osoba.
  8. Lakše mi je da komuniciram licem-u-lice nego onlajn.
  9. Teško mi je da budem to što jesam u realnom svetu, van interneta.
  10. Imam utisak da sam ista osoba na internetu i u realnom svetu.
  11. Više volim da sam onlajn, nego oflajn.
  12. U onlajn okruženju stalno se predstavljam kao više različitih osoba.
  13. Kad sam onlajn mogu da pobegnem od sebe.
  14. Često glumim da sam različita osoba na određenim onlajn platformama.
  15. Onlajn okruženje mi dozvoljava da kreiram nov identitet.
  16. Onlajn lakše mogu da pokažem svoje najbolje kvalitete.
  17. Onlajn mogu da pričam s ljudima koji inače ne bi pričali sa mnom uživo.
  18. Drugačija sam osoba u zavisnosti od toga na kojoj sam onlajn platformi.
  19. Mnogo mi je prijatnije da se ponašam onako kako želim kada sam onlajn.
  20. Uživam da glumim različite identitete onlajn.
  21. Osećam kao da onlajn mogu da budem idealna verzija sebe.

KEY / KLJUČ:

Ideal self / Idealistična samoprezentacija: 1, 4, 5, 7, 13, 16, 17, 19, 21

Multiple self / Višestruka samoprezentacija: 12, 14, 15, 18, 20

Consistent self / Usklađena samoprezentacija: 2(R), 3, 6, 10

Online presentation preference / Preferncija onlajn samoprezentacije: 8(R), 9, 11
